# Expansion and Evolutionary Patterns of Glycosyltransferase Family 8 in Gramineae Crop Genomes and Their Expression under Salt and Cold Stresses in *Oryza sativa* ssp. *japonica*

**DOI:** 10.3390/biom9050188

**Published:** 2019-05-15

**Authors:** Weilong Kong, Ziyun Gong, Hua Zhong, Yue Zhang, Gangqing Zhao, Mayank Gautam, Xiaoxiao Deng, Chang Liu, Chenhao Zhang, Yangsheng Li

**Affiliations:** State Key Laboratory for Hybrid Rice, College of Life Sciences, Wuhan University, Wuhan 430072, China; Weilong.Kong@whu.edu.cn (W.K.); Gziyun@whu.edu.cn (Z.G.); zhonghua0103@whu.edu.cn (H.Z.); Yue.Zhang-@whu.edu.cn (Y.Z.); zhaogangqing@whu.edu.cn (G.Z.); mayankgautam@whu.edu.cn (M.G.); 2017102040003@whu.edu.cn (X.D.); lchang@whu.edu.cn (C.L.); zch_nx@126.com (C.Z.)

**Keywords:** GT8, glycosyltransferase family, Gramineae, gene duplication, evolutionary patterns, qRT-PCR, cold stress, salt stress

## Abstract

Plant cell walls play a fundamental role in several ways, providing structural support for cells, resistance against pathogens and facilitating the communication between cells. The glycosyltransferase family 8 (GT8) is involved in the formation of the plant cell wall. However, the evolutionary relationship and the functional differentiation of this important gene family remain obscure in Gramineae crop genomes. In the present investigation, we identified 269 GT8 genes in the seven Gramineae representative crop genomes, namely, 33 in *Hordeum vulgare*, 37 in *Brachypodium distachyon*, 40 in *Oryza sativa* ssp. *japonica*, 41 in *Oryza rufipogon*, 36 in *Setaria italica*, 37 in *Sorghum bicolor*, and 45 in *Zea mays*. Phylogenetic analysis suggested that all identified GT8 proteins belonged to seven subfamilies: galacturonosyltransferase (GAUT), galacturonosyltransferase-like (GATL), GATL-related (GATR), galactinol synthase (GolS), and plant glycogenin-like starch initiation proteins A (PGSIP-A), PGSIP-B, and PGSIP-C. We estimated that the GAUT subfamily might be further divided into four subgroups (I–IV) due to differentiation of gene structures and expression patterns. Our orthogroup analysis identified 22 orthogroups with different sizes. Of these orthogroups, several orthogroups were lost in some species, such as *S. italica* and *Z. mays*. Moreover, lots of duplicate pairs and collinear pairs were discovered among these species. These results indicated that multiple duplication modes led to the expansion of this important gene family and unequal loss of orthogroups and subfamilies might have happened during the evolutionary process. RNA-seq, microarray analysis, and qRT-PCR analyses indicated that GT8 genes are critical for plant growth and development, and for stresses responses. We found that *OsGolS1* was significantly up-regulated under salt stress, while *OsGAUT21*, *OsGATL2*, and *OsGATL5* had remarkable up-regulation under cold stress. The current study highlighted the expansion and evolutionary patterns of the GT8 gene family in these seven Gramineae crop genomes and provided potential candidate genes for future salt- and cold- resistant molecular breeding studies in *O. sativa*.

## 1. Introduction

Glycosyltransferases (GTs) (EC 2.4.x.y), a large superfamily of enzymes, catalyze the transfer of sugar moieties from activated donor molecules to specific acceptor molecules by forming glyosidic bonds, which are found to be involved in the biosynthesis of disaccharides, oligosaccharides, and polysaccharides [1,2]. At present, GTs are subdivided into 106 families [3,4]. Among them, those of glycosyltransferase family 8 (GT8) have genes which might be involved in plant cell wall biosynthesis and modification [5]. According to molecular evolutionary analysis, GT8 was divided into seven subfamilies: galacturonosyltransferase (GAUT), galacturonosyltransferase-like (GATL), GATL-related (GATR), galactinol synthase (GolS), and plant glycogenin-like starch initiation proteins A (PGSIP-A), PGSIP-B, and PGSIP-C [6].

*GT8* genes are present in bacteria, animals, fungi, and plants [6]. GT8 proteins can use UDP-glucose, UDP-galactose, UDP-xylose, UDP-galacturonic acid, or UDP-glucuronic acid as donors [1]. GT8 proteins participate in the biosynthesis of glycoproteins [7,8], lipopolysaccharides [9], glycogen [10], plant cell walls [11], and small oligosaccharides [12]. In *Arabidopsis*, GT8 family contains 41 proteins belonging to four major subfamilies: GAUT, GATL, GolS, and PGSIP [6]. Among them, AtGAUT1 and AtGAUT7 proteins can synthesize the pectic polymer homogalacturonan [11]. It has been reported that *Arabidopsis* GAUT proteins are involved in the biosynthesis of pectin and xylan of the cell walls and seed testa [13]. Moreover, three members from *Arabidopsis* stress-responsive GolS subfamily were shown to synthesize galactinol by transferring galactose onto inositol. Additionally, *AtGolS1* and *AtGolS2* were triggered by drought and high-salinity stresses, but not by cold stress. However, *AtGolS3* was shown to be induced by cold stress but not by drought or salt stress [12]. Another study reported that stress-inducible GolS proteins played an essential role in plant abiotic stress tolerances via the accumulation of galactinol and raffinose acting as osmoprotectants [12]. *Arabidopsis* PGSIP proteins were reportedly associated with the synthesis of primers significant for starch synthesis [6,14]. In rice, 40 GT8 genes were firstly identified and grouped into seven subfamilies [6]. It is reported that CRISPR-Cas9 knockout of a *GT8* gene (*Os10g0555100*) can regulate dwarfing and spike-shaped modifications in rice [15].

Whole genome duplication (WGD), segmental duplication, and tandem duplication are well known to play important roles in structural changes of genome and functional diversification of genes [16,17]. Several studies have demonstrated that almost all plants have experienced WGD events [17,18]. For example, *Arabidopsis* has experienced multiple WGDs during the evolutionary process [19,20,21], and grass genome and major dicotyledonous plants shared a hexaploid ancestor and experienced a WGD about 130–150 million years ago (Mya) [22]. Also, genomes of Gramineae plants experienced three WGDs, and recent WGDs occurred about 70 Mya [23,24]. In addition to WGD, segmental duplication and tandem duplication also play an essential role in the expansion of gene families [25]. As such, 16.2% and 16.5% of all genes in *Arabidopsis* and rice were identified as tandem duplicates [25]. Theoretical models of gene family evolution proposed that gene families continuously undergo stochastic gain and loss events and these processes are related to functional fates of gene duplicates from various duplication events [26,27,28]. Current hypotheses associated with fate of gene duplication and divergences proposed that most novel duplicated copies are randomly lost through recombination-dependent delectation and a few duplicated copies can be preserved in the form of processed pseudogenes owing to the accumulation of loss-of-functional mutations [29,30]. Alternatively, new copies are fixed and subsequently preserved by selection for new functions (NF, neofunctionalization), partitioning of the original functions (SF, subfunctionalization), or SF followed by NF (SNF, subneofunctionalization) [30,31,32,33,34,35].

Comparative analysis of closely related lineages is an efficient strategy to gain a better understanding of the evolutionary dynamics of a gene family and its consequences. Gramineae are composed of several crops of high economic and industrial value, with well-characterized phylogeny and numerous genetic resources. Gramineae therefore are an exceptional model system for the study of short-term evolutionary dynamics of gene families in the plants. On the other hand, the evolutionary dynamic of the GT8 gene family in the Gramineae is poorly documented at present. In addition, salt and cold stresses are the two major threats to rice growth and yield [36,37,38]. To our knowledge, the expression responses of rice GT8 genes under these two stresses have not been well-studied to date. In this study, we used bioinformatics approaches to identify GT8s by screening seven Gramineae crops’ genomes, namely, *Brachypodium distachyon*, *Hordeum vulgare*, *Setaria italica*, *Sorghum bicolor*, *Zea mays*, *Oryza rufipogon*, and *Oryza sativa* ssp. *japonica*. We then characterized the phylogenetic relationship, chromosomal location, gene structure, protein motifs, promoter Cis-elements, orthogroups, duplication events, and microsynteny relations. Furthermore, expression patterns of this family in rice were analyzed to characterize functional differentiation of rice GT8 genes and to select salt- or cold-responsive GT8 genes. We explored the expansion and evolutionary patterns of GT8 in the selected Gramineae crops and provided a novel insight into the functions of rice GT8 (*OsGT8*) genes under cold and salt stresses.

## 2. Materials and Methods

### 2.1. Plant Materials

Rice ‘Nipponbare’ (*O. sativa* ssp. *japonica*) was used for the quantitative real-time RT-PCR (qRT-PCR) analysis. After 2 days of germination in water at 37 °C, seeds were grown in containers with sponges as supporting materials in Yoshida solution for further growth. All seedlings were grown with 60% relative humidity and with a daily photoperiod of 14 h light 30 °C/10 h dark 22 °C [36,37]. Three-leaf stage seedlings were transferred to 200 mM NaCl Yoshida solution for analyzing salt stress [37]. For cold stress analysis, growth temperature was changed to 4 °C. Samples (leaves) were collected at 0, 3, 12, and 24 h for RNA extraction. Three biological replicates were used, each of which was collected from 15 seedlings. Total RNA was extracted using TRIzol method (Invitrogen, Beijing, China) and reverse transcribed into cDNA using the PrimeScript RT reagent Kit (TakaRa, Dalian, China).

### 2.2. Identification of the GT8 Gene Family in Gramineae Crop Genomes

Genome datasets containing the protein and cDNA sequences of *O. sativa* ssp. *japonica* were downloaded from MSU 7.0 (http://rice.plantbiology.msu.edu). Genome datasets of *B. distachyon* (v3.0), *H. vulgare* (IBSC_v2), *S. italica* (v2.0), *S. bicolor* (NCBIv3), *Z. mays* (B73_RefGen_v4), and *O. rufipogon* (OR_W1943) were downloaded from EnsemblPlants (http://plants.ensembl.org/index.html). Rice GT8 proteins were obtained according to previously identified results [6]. The Hidden Markox Model (HMM) profile of the Glyco_transf_8 domain (PF01501) was obtained from Pfam (http://pfam.xfam.org/). First, rice GT8 proteins were used to search GT8 proteins in protein datasets of *O. sativa* ssp. *japonica*, *B. distachyon*, *H. vulgare*, *S. italica*, *S. bicolor*, *Z. mays*, and *O. rufipogon* using BlastP method with E-value cut off e-5 (ftp://ftp.ncbi.nlm.nih.gov/blast/executables/blast+/LATEST) [39]. Simultaneously, the Glyco_transf_8 domain was utilized to investigate these seven species’ protein datasets for GT8 proteins using HMMER 3.0 software (http://hmmer.org/) [40]. The search results of the two methods were collected and we selected only the longest protein sequence from one gene for further analysis. Sequences with ‘X’ >10 bp were also excluded from further analysis. Finally, Glyco_transf_8 domains of all remaining sequences were checked using SMART (http://smart.embl-heidelberg.de/) and Pfam (http://pfam.xfam.org/search/sequence) [39,40].

### 2.3. Multiple Sequence Alignments and Phylogenetic Analysis of GT8 Proteins in Gramineae Crop Genomes

All identified GT8 proteins were aligned by MAFFT version 7 with G-INS-1 progressive methods and other default parameters (https://mafft.cbrc.jp/alignment/server/) [41,42]. Then, a UPGMA phylogeny tree of all proteins was generated with default parameters in the similar website. Finally, the phylogeny tree was visualized by using MEGA 6.0 (https://www.megasoftware.net/).

### 2.4. Orthogroups Analysis (Orthologues) of All GT8 Genes in Gramineae Crop Genomes

Firstly, an all-vs-all BlastP search was performed by diamond software with parameters Evalue 1e-3 (https://ab.inf.uni-tuebingen.de/software/) [43] as the input file for OrthoFinder software [44]. Afterwards, orthogroups were analyzed according to previously described method [44]. Moreover, the phylogenetic tree of all tested species was made based on the result of orthogroups using STAG and STRIDE algorithms in OrthoFinder software [44].

### 2.5. Gene Structures and Conserved Motifs

Genomic structures of GT8 genes were analyzed by GFF3 files and conserved motifs of all GT8 proteins were obtained using the MEME program (http://meme-suite.org/tools/meme) with the following parameters: 12 motifs, motif width between 6 and 100, and other default parameters [39]. Then, TBtools was used to visualize the phylogenetic tree, gene structure, and conserved motifs of GT8 genes [37,45].

### 2.6. Chromosomal Locations, Gene Duplication Events, and Microsynteny Analysis of GT8 Proteins in Gramineae Crop Genomes

GT8 genes in all tested species were assigned to chromosomes according to site information from GFF3 files. Within each species, gene duplication events of the GT8 gene family were analyzed by the ‘duplicate_gene_classifier’ script in MCScanX with an E-value of 1e^−5^ in BlastP search [37,46]. Chromosomal locations and gene duplication events were visualized by Perl-based tool, Circos (http://circos.ca/) [37,47]. The synonymous (Ks) and nonsynonymous (Ka) substitution rates were calculated using DnaSP 5.0 (http://www.ub.edu/dnasp/) [48]. Divergence times of all duplicate pairs were estimated by using T = Ks/(2 × 9.1 × 10^−9^) × 10^−6^ Mya [37,49]. The microsynteny relations of *O. sativa* ssp. *japonica* with *B. distachyon*, *H. vulgare*, *S. italica*, *S. bicolor*, *Z. mays*, and *O. rufipogon* were analyzed by using Multiplae Collinearity Scan toolkit X version (MCScanX) as described previously [46]. Then, collinearity pairs of GT8 genes in *O. sativa* ssp. *japonica* and the other six species were visualized by the ‘dual synteny plotter’ in TBtools [45].

### 2.7. Analysis of Cis-elements and Prediction of Subcellular Localizations of GT8 Genes in O. sativa ssp. japonica

Cis-acting regulatory elements (Cis-elements) in promoter regions (2 Kbp upstream from the translation start site, ATG) of the rice GT8 genes were identified using the PLANTCARE database (http://bioinformatics.psb.ugent.be/webtools/plantcare/html) [50]. The subcellular localizations of GT8 proteins were estimated by using Plant-mPLoc server (http://www.csbio.sjtu.edu.cn/bioinf/plant-multi/) [39]. Transmembrane helical domains (TMHs) were predicted by using TMHMM Severv.2.0 (http://www.cbs.dtu.dk/services/TMHMM/).

### 2.8. Expression Patterns and Coexpression Analysis of GT8 Genes in O. sativa ssp. japonica

Raw datasets of different tissues of rice ‘Nipponbare’ (*O. sativa* ssp. *japonica*) relating to leaves at 20 days, post-emergence inflorescence, pre-emergence inflorescence, anthers, pistils, seeds 5 days after pollination (DAP), embryos at 25 DAP, endosperm at 25 DAP, seeds at 10 DAP, shoots, and the seedling four-leaf stage (SRX100741, SRX100757, SRX100743, SRX100745, SRX100746, SRX100747, SRX100749, SRX100753, SRX100754, SRX100756, SRX100755, SRR042529, and SRX016110) were downloaded from https://www.ncbi.nlm.nih.gov/, and analyzed according to a previously described protocol [46].

The microarray datasets of cold stress (GSE57895, 96 microarray datasets,) [51] and salt stress (GSE76613, 96 microarray datasets) were obtained from the Gene Expression Omnibus (GEO, https://www.ncbi.nlm.nih.gov/geo/) database and analyzed according to our previously described method [36]. The cold-stressed samples (shoots and roots) were collected from three-leaf stage seedlings of two rice subspecies, TNG67 (*indica*) and TCN1 (*japonica*), at 0 h, 3 h, and 24 h for 4 °C and for recovered material after 24 h. [51]. Similarly, the salt-stressed samples (shoots and roots) were collected from three-leaf stage seedlings of two rice subspecies, TNG67 (*indica*) and TCN1 (*japonica*), at 0 h, 3 h, 24 h, and recovery 24 h after 250 mM NaCl treatment [36]. Detailed information (experimental design, array information, data processing, and platform ID) of these two microarray datasets can be obtained from the GEO by searching for registration ID (GSE76613 and GSE57895). Co-expression analysis of OsGT8 genes was conducted using Pearson’s correlation coefficient based on the expression matrix from microarray analysis and RNA-seq results of the present study [37,49].

### 2.9. Quantitave Real-Time PCR (qRT-PCR)

Rice salt-responsive (*OsGolS1*) or cold-responsive GT8 genes (*OsGAUT21*, *OsGATL2*, and *OsGATL5*) from microarray analyses were verified by using qRT-PCR. Primers of these four rice GT8 genes were designed by Primer 5.0 (Table S1). The qRT-PCR reaction (10 μL) was formulated using the 2 X SYBR Green qPCR Master Mix (US Everbright^®^ Inc., Suzhou, China). All qRT-PCRs were carried out on a CFX96 Touch™ Real-Time PCR Detection System (Bio-Rad, Hercules, CA, USA). The *actin* gene was employed as an internal control [37]. Three biological replicates (from three independent RNA samples) were used for qRT-PCR. For each biological replicate, three technical replicates were also used. The average threshold cycle (Ct) from three biological replicates was employed to calculate the gene expression fold change by the 2^−ΔΔCT^ method [37,49].

## 3. Results

### 3.1. Identification and Classification of GT8 Genes in Gramineae Crop Genomes

**Figure 1:**
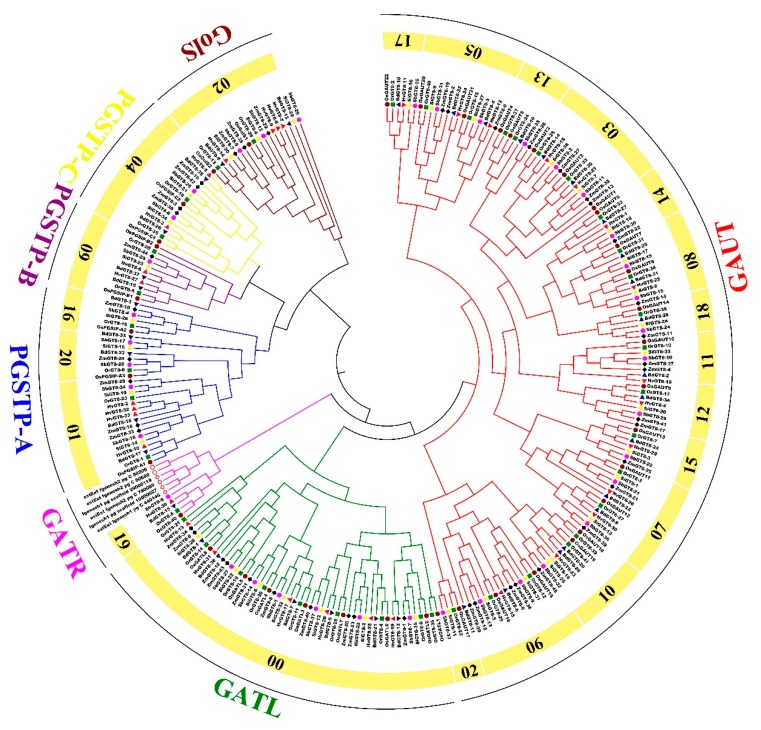
A UPGMA phylogeny tree of glycosyltransferase family 8 (GT8) protein sequences from *Hordeum vulgare*, *Brachypodium distachyon*, *Oryza sativa* ssp. *japonica*, *Oryza rufipogon*, *Setaria italica*, *Sorghum bicolor*, and *Zea mays*. Different colors of circles represent different subfamilies. The numbers in yellow circles represent different orthogroups, such that 00 means Orthogroup0. The different species are displayed by different shaped markers. Protein* is not in any orthogroups. A high-resolution version of Figure 1 is provided in Figure S1.

**Figure 2:**
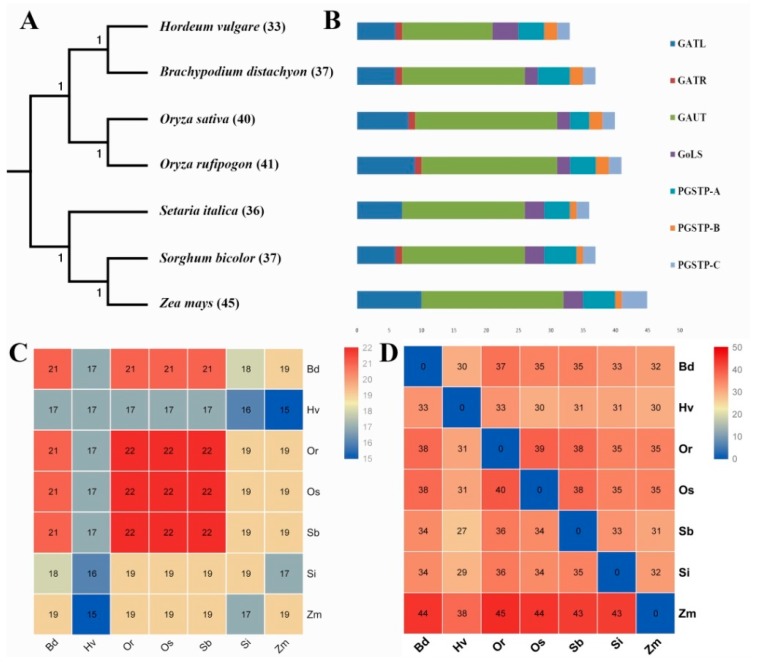
The numbers of GT8 genes/orthogroups/orthologues. (**A**) The phylogenetic tree of these tested species was constructed based on the result of orthogroups using STAG and STRIDE algorithms in OrthoFinder software [44]. (**B**) Histogram charts of different subfamilies in *H. vulgare*, *B. distachyon*, *O. sativa* ssp. *japonica*, *O. rufipogon*, *S. italica*, *S. bicolor*, and *Z. mays*. (**C**) The numbers of GT8 orthogroups among these seven species. (**D**) The numbers of GT8 orthologues among these seven species. The vertical color scale at the right of Figure 2 (**C**,**D**) represents GT8 gene number from lower (blue color) to higher (red color).

Using BlastP similarity and HMM searches, a total of 269 members of GT8 gene family were identified in *H. vulgare* (33), *B. distachyon* (37), *O. sativa* ssp. *japonica* (40), *O. rufipogon* (41), *S. italica* (36), *S. bicolor* (37), and *Z. mays* (45), respectively (Figure 1 and Figure 2, Table S2). We found that *Z. mays* had more GT8 genes than other tested species; the number of GT8 genes in *H. vulgare* was lower than that in other tested species; the numbers of GT8 genes in the remaining species were similar (37–41), especially in *O. sativa* ssp. *japonica* (40), and *O. rufipogon* (41) (Figure 2, Table S2). To reveal the quantity difference of GT8 genes in these species, subfamily classifications and orthogroup identification were conducted and results showed that all GT8 proteins grouped into seven subfamilies, namely GTAL, GATR, GAUT, GoLS, PGSIP-A, PGSIP-B, and PGSIP-C (Figure 1, Table S2). The number of GTAL, GATR, and GAUT subfamilies led to major number difference in GT8 genes among these species. For instance, there were 10, 9, and 8 GATL genes in *Z. mays*, *O. rufipogon*, and *O. sativa* ssp. *japonica*, while there were only 6, 7, 6, and 6 GATL genes in *S. italica*, *S. bicolor, B. distachyon*, and *H. vulgare*, respectively. There were 19–22 GAUT genes in *B. distachyon*, *O. sativa* ssp. *japonica*, *O. rufipogon*, *S. italica*, *S. bicolor*, and *Z. mays*, whereas there was only 14 GAUT genes in *H. vulgare* (Figure 2). Orthogroups result strongly supported our phylogeny tree and displayed a total of 22 orthogroups among these seven species, namely, 21 in *B. distachyon*, 17 in *H. vulgare*, 22 in *S. bicolor*, *O. sativa* ssp. *japonica*, and *O. rufipogon*, and 19 in *S. italica* and *Z. mays* (Figure 2C and Table S3). The paralogue numbers in these orthogroups were different. For example, Orthogroup00 was the largest orthogroup, while Orthogroup 14–21 belonged to single orthogroups (Tables S3 and S4). Additionally, the numbers of orthologues were also different among these species (Figure 2D, Tables S5–S11). These results indicated that ancestors of Gramineae might contain 19 GT8 orthogroups and expansions of the GT8 family, and unequal losses of different orthogroups might have occurred during the Gramineae species differentiation process.

### 3.2. Expansion Pattern of GT8 Genes in Gramineae Crop Genomes

**Figure 3:**
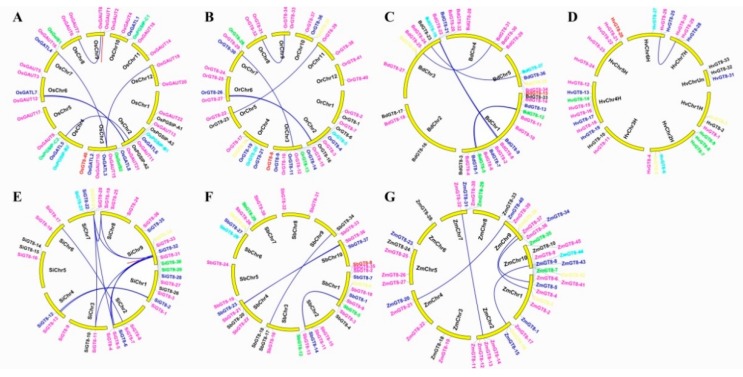
The chromosome location and duplication events of GT8 genes in seven species, namely, *O. sativa* ssp. *japonica* (**A**), *O. rufipogon* (**B**), *B. distachyon* (**C**), *H. vulgare* (**D**), *S. italica* (**E**), *S. bicolor* (**F**), and *Z. mays* (**G**). The blue lines represent whole genome duplication (WGD)/segmental duplication and red lines mean tandem duplication events. The different color genes belong to different subfamilies. A high-resolution version of Figure 3 is provided as Figure S2.

To understand the expansion mechanism of paralogues, we investigated gene duplication modes within each species. Among all tested species, GT8 genes were unevenly distributed on all chromosomes (Chrs) (Figure 3). For example, nine GT8 genes were located on rice Chr3, of which only one GT8 gene was located on rice Chr5 in *O. sativa* ssp. *japonica* (Figure 3A). We found WGD/segmental duplications were the major gene duplication modes in these tested species (Figure 3A–G). Furthermore, tandem duplication also played an important role in the expansion of the GT8 family in *O. sativa* ssp. *japonica* and *S. italica* (Figure 3A,E). The numbers of WGD/segmental duplications differed among these species. There were 7, 5, 6, 1, 13, 5, and 5 duplication pairs in *O. sativa* ssp. *japonica*, *O. rufipogon*, *B. distachyon*, *H. vulgare*, *S. italica*, *S. bicolor*, and *Z. mays* (Figure 3), respectively. We also noticed that the number of duplication pairs was less than the number of paralogues (i.e., no duplication event in paralogues from orthogroup00), indicating that other duplication modes also play significant roles in the expansion of GT8 family, such as proximal, dispersed, and replicative transposition. Interestingly, although the numbers of duplication events vary, the total number of GT8 genes was close among these species (except *Z. mays*). Based on these results, it can be deduced that multiple duplication modes play an essential role in the expansion of GT8 family in Gramineae crop genomes and the principal duplication mode might be different between these tested species.

The Ka/Ks ratios of all duplicate gene pairs were less than 1, illustrating that all duplicate gene pairs were under a strong negative selection during the Gramineae evolutionary process (Table S12). Divergence times were ranged from 1.00 to 185.04 Mya (Table S12). These results indicated that duplication events play a central role in the expansion of GT8 family during a long-term evolutionary process.

### 3.3. Collinearity Relations of O. sativa ssp. japonica with Other Tested Species

**Figure 4:**
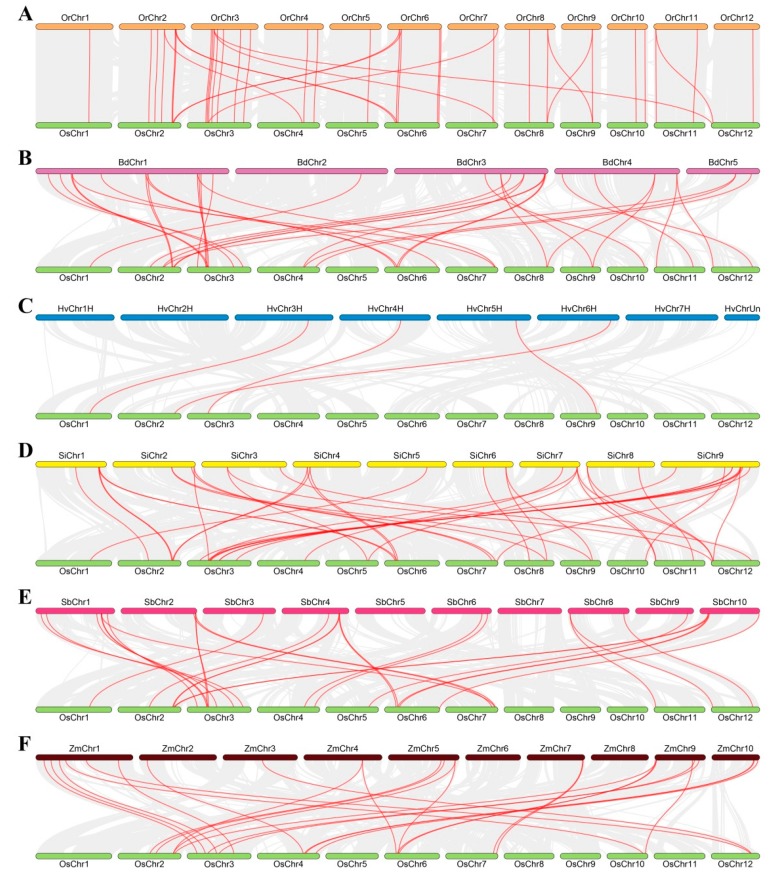
Collinearity relationships of GT8 genes in *O. sativa* ssp. *japonica* and *O. rufipogon* (**A**), *B. distachyon* (**B**), *H. vulgare* (**C**), *S. italica* (**D**), *S. bicolor* (**E**), and *Z. mays* (**F**), respectively.

To elucidate the evolutionary origins of GT8 genes within Gramineae, the molecular phylogeny of the GT8 family was analyzed by comparative genome analysis using MCScanX toolkit. We found 40, 35, 4, 39, 28, and 25 collinear gene pairs between *O. sativa* ssp. *japonica* and *O. rufipogon*, *B. distachyon*, *H. vulgare*, *S. italica*, *S. bicolor*, and *Z. mays*, respectively. The collinearity relationships of GT8 genes were strongly conserved between *O. sativa* ssp. *japonica* with *B. distachyon*, *O. rufipogon*, *S. bicolor*, *S. italica*, and *Z. mays*, while GT8 genes showed weak collinearity relationship between *O. sativa* ssp. *japonica* and *H. vulgare* (Figure 4). This finding exhibited closer relationship between *O. sativa* ssp. *japonica* and *B. distachyon*, *O. rufipogon*, *S. bicolor*, *S. italica*, and *Z. mays*, which supported their evolutionary distance.

### 3.4. Sequence Characteristics and Intron Number Analyses

We observed that the members from the same subfamily among these seven species showed similar motifs distributions and compositions (Figure S3), suggesting that functions of GT8 genes were conserved among these species. We also found that introns displayed similar patterns and distributions (Figure S3), indicating that introns were relatively conserved during the evolution in the Gramineae GT8 gene family. Remarkably, we noticed that GAUT subfamily displayed conserved motifs distributions, while this subfamily appeared to be classified into four groups within genomic structure (Figure S3) namely GAUT I, GAUT II, GAUT II, and GAUT IV.

### 3.5. Subcellular Localization and Cis-Elements Predicton

The great majority of *O. sativa* ssp. *japonica* proteins in GATL and GAUT subfamilies were predicted to have one TMH (Table S13). Members of GolS and PGSIP-A had no TMH, whereas members of PGSIP-B and PGSIPC had 1–6 TMHs (Table S13). We found that most GT8 proteins were located on the chloroplast, while several proteins were located on the nucleus, cell membrane, and peroxisome (Table S13).

PlantCare database showed a high frequency of occurrence of Cis-elements in OsGT8 genes and 45 Cis-elements were identified (Figure S4, Table S14). Based on the published description [37], these Cis-elements can be divided into three primary categories and 19 secondary categories (Figure S4A,C). Among them, the growth and development primary category showed a higher frequency of Cis-element occurrence than the stress response and phytohormone response primary category (Figure S4C). We found that the top two secondary categories were the abscisic acid responsiveness (ABRE) and the MeJA responsiveness categories (CGTCA-motif and TGACG-motif) in phytohormone response primary category; the top category was the light responsiveness category in the growth and development primary category; the top three categories were anoxic-specific inducibility (ARE and GC-motif), drought inducibility (MBS), and low temperature responsiveness categories (LTR) in the stress response primary category (Figure S4A). The result of Cis-element positions showed that Cis-elements were unevenly distributed on promoters of all OsGT8 genes (Figure S4B). On the present finding basis, we proposed that OsGT8 genes might be significant to improve rice stress tolerances.

### 3.6. Coexpression Relation of OsGT8 Genes

Expression analysis of genes can provide vital clues to their functions. We thus analyzed public available RNA-sequencing data and microarray data from NCBI (https://www.ncbi.nlm.nih.gov/) according to the previously described method [36,37]. Pearson’s correlations showed that some GT8 genes had positive relations (>0.5) in all tested tissues, under cold stress or salt stress (Figure S5). However, the co-expression relations were different in all the tested tissues, under cold stress or salt stress (Figure S5). These results suggested that GT8 genes might function in the growth and abiotic stress responses by regulating their own expressions, while their interaction modes are extremely different under different conditions.

It is well known that duplicate genes face different fates after duplication events [26,27,28]. Previous studies proposed that r <0.3, 0.3 < r < 0.5, and r > 0.5 mean divergent, ongoing divergent, and non-divergent, respectively [37,49]. We found that four duplicate gene pairs were lower than 0.3, namely, *OsGAUT19*–*OsGAUT15*, *OsGAUT11*–*OsGAUT12*, *OsGAUT7*–*OsGAUT8*, and *OsGAUT1*–*OsGAUT2* (Table S15), which indicated that these gene pairs were divergent. One gene pair (*OsGAUT15*–*OsGAUT16*) was more than 0.5 (Table S15), indicating *OsGAUT15* and *OsGAUT16* were non-divergent. Interestingly, *OsGATL6* and *OsGATL7* were 0.11 in all tissues, while 0.73 and 0.53 in cold and salt stress (Table S15). This revealed that *OsGATL6* and *OsGATL7* could belong to subfunctionalization or subneofunctionalization.

### 3.7. Expression Patterns of OsGT8 Genes

**Figure 5:**
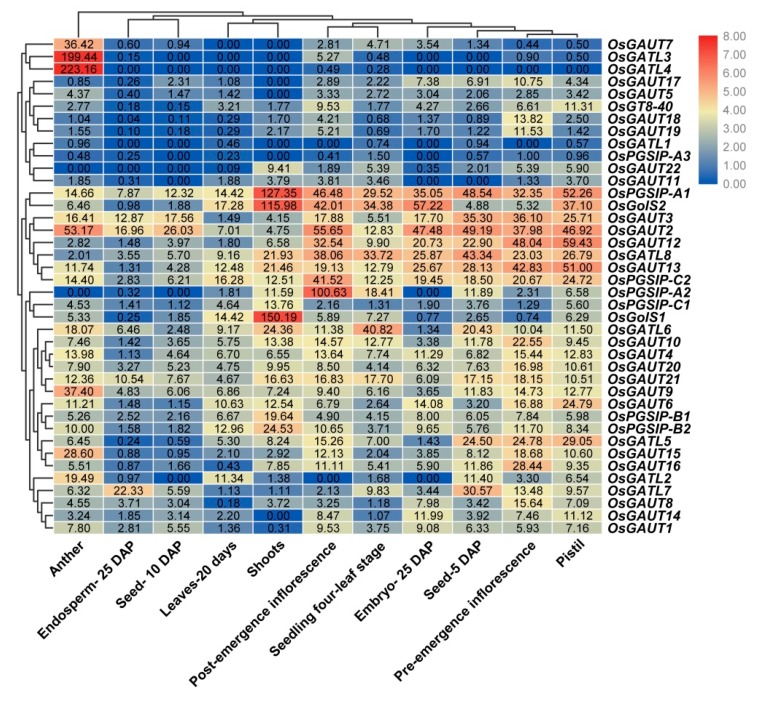
Expression profiles of OsGT8 genes in different tissues. The values in the color scale represent log_2_^FPKM^: red/blue indicates high level/low level of transcript abundance.

In this study, we found that most OsGT8 genes were expressed in all tissues with different levels (Figure 5). Some genes showed relatively high levels in one or several tissues (Figure 5). For instance, *OsGATU7*, *OsGATL3*, and *OsGATL4* were highly expressed in anthers; *OsGolS1* had high expression level in shoots; *OsGATL6* were strongly expressed in seedling at the four-leaf stage; *OsGAUT4*, *OsGAUT20*, *OsGAUT21*, and *OsGAUT9* had high expression levels in pre-emergence inflorescence; and *OsPGSIP-A1*, *OsGolS2*, *OsGAUT3*, *OsGAUT2*, *OsGAUT12*, *OsGATL8*, *OsGAUT13*, and *OsPGSIP-C2* were highly expressed in multiple tissues and stages.

**Figure 6:**
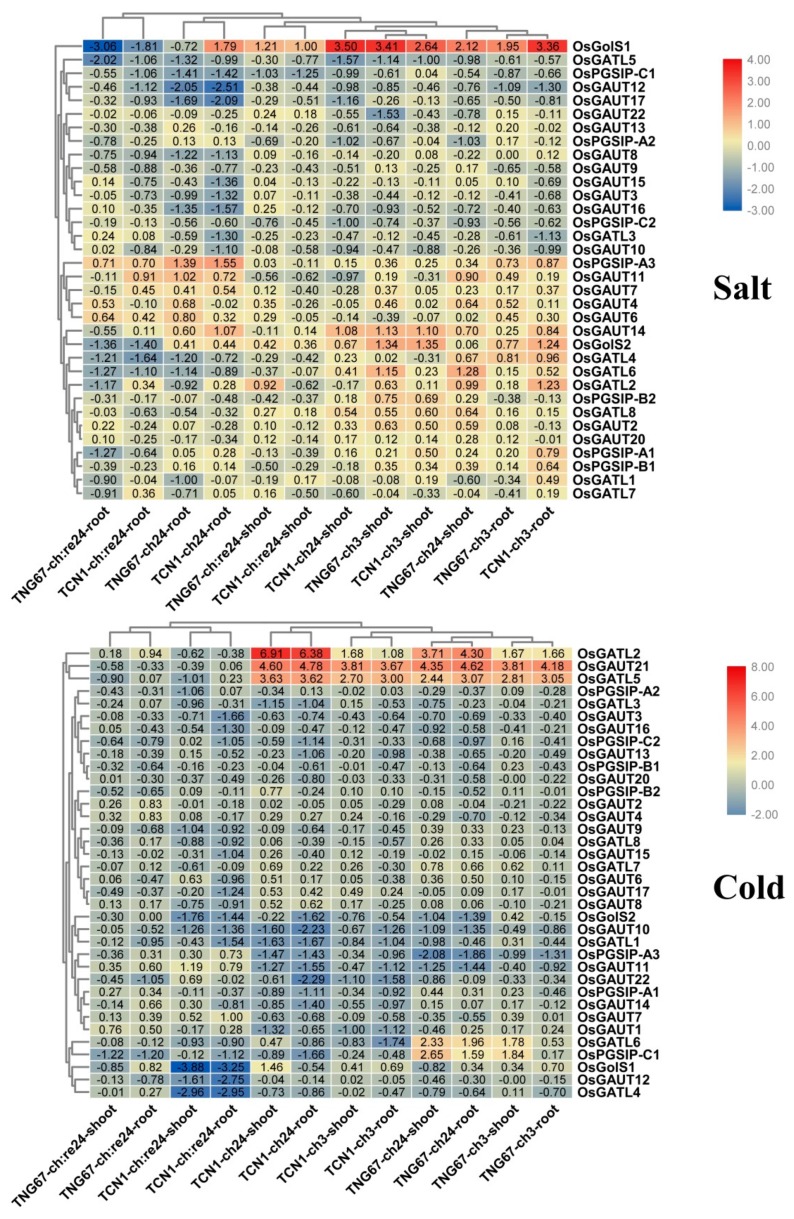
Expression profiles of OsGT8 genes under salt and cold stress. TNG67, TCN1 indicate two rice cultivars. ch3, ch24, ch:re24 represent 3 h after treatment, 24 h after treatment, and subsequent 24 h recovery, respectively. The values in the color scale represent log_2_^foldchange^: normalization to the control and with significant results for the *t*-test (*p*-value < 0.05) based on six replicates (3 biological repeats × 2 technical repeats) for each treatment compared with the control [36].

**Figure 7:**
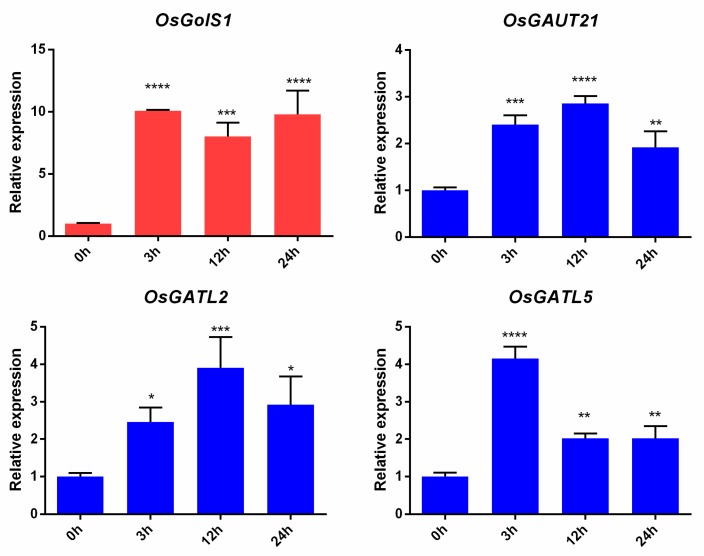
The qRT-PCR of the salt/cold-responsive GT8 genes in ‘Nipponbare’ seedling leaf after NaCl treatment/Cold (4 °C) treatment for 0 h, 3 h, 12 h, and 24 h. Red indicates salt stress and blue indicates cold stress. The fold changes of these genes were calculated by 2^−ΔΔCT^ method from three biological replicates. The bars show the standard deviations of three biological replicates and * represents a significant difference relative to the 0 h group (* *p* < 0.05, ** *p* < 0.01, *** *p* < 0.001, **** *p* < 0.0001).

Under salt stress, the majorities of GT8 genes were down-regulated (Figure 6), while *OsGolS1* was up-regulated in root and shoot at almost all tested time points and showed down-regulation in root during the recovery process (Figure 6). Three GT8 genes (*OsGATL2*, *OsGAUT21*, and *OsGATL5*) were up-regulated in roots and shoots under cold stress, while they all were down-regulated during the recovery process (Figure 6). To further verify the accuracy of the results, these stress-responsive genes were further analyzed by using qRT-PCR. The qRT-PCR analysis also supported that *OsGolS1* was a salt-responsive gene and *OsGATL2*, *OsGAUT21*, and *OsGATL5* were cold salt-responsive genes, indicating that these four genes might play crucial roles in rice abiotic stress responses (Figure 7).

## 4. Discussion

In the present study, 269 GT8 genes from seven Gramineae crops were identified. We concluded that no direct relevance between genome sizes and the number of GT8 genes. For example, there were 37 GT8 genes in *B. distachyon* (genome size: 355 Mbp) [52], while there were 33 genes in *H. vulgare* (4.79 Gbp) [53]. Moreover, there was no significant difference in the number of GT8 genes in *S. italica* (number: 36) and *S. bicolor* (37), whereas the genome sizes were obviously different (490 Mbp and 730 Mbp) [54,55]. In addition, there was direct relationship between WGDs and the number of GT8 genes. For example, *Z. mays* had a greater GT8 genes (number: 45) than other tested species (33–41), which resulted from the fact that *Z. mays* experienced a specific WGD on the basis of three WGDs of all Gramineae plants [56]. Strikingly, only 19 orthogroups were identified among these species, whereas there were 33–41 genes in the remaining species. Many duplication events were found among these species and divergence times ranged from 1.00 to 185.04 Mya. These results indicated that ancestors of Gramineae might contain 19 GT8 orthogroups and the new genes were acquired due to different gene duplication modes in Gramineae. Based on subfamily classification and orthogroups results, we proposed that the differentiation of subfamilies had been completed in the ancestors of Gramineae. However, orthogroups and even subfamilies were lost in some species during the evolutionary process. For instance, five orthogroups were lost in *H. vulgare*; three orthogroups were lost in *S. italica* and *Z. mays*; and the whole GATR subfamily was lost in *S. italica* and *Z. mays*. These pieces of evidence indicated that members of GT8 family have functional redundancy and the loss of individual genes and subfamilies does not have a significant negative impact on plants. However, this speculation needs further verification. Interestingly, further analyses of orthogroups revealed that the different expansion modes in all identified orthogroups, while expansion patterns (numbers) of these orthogroups among these species were similar. This result suggested that Gramineae crops were under a similar selection direction during the evolutionary process.

In contrast to Yin et al. [6], we identified one new *OsGT8* gene (*LOC_Os03g56620.1* belongs to GATR subfamily, named *OsGT8-40*) in rice. Noticeably, diverse expression patterns and differentiated gene structural arrangements supported that the function of GAUT subfamily members might be different. We thus classified them into four (I–IV) subgroups. Consistent with the previous investigation [37,57], the present result also suggested that tandem and WGDs/segmental duplication events play essential roles in the expansion of GT8 gene family in Gramineae crops. In addition, Pearson’s correlation coefficient investigation exhibited that duplicate rice GT8 gene pairs might have showed functional differentiations. Our data is preliminary with respect to the functional differentiation; further investigations are required in order to make conclusions.

Until now, increasing evidence on distinct species has confirmed that GT8 genes are critical for plant growth and development [1,6,12,13,14]. For example, *Arabidopsis* GAUT proteins are involved in the biosynthesis of pectin and xylan in cell walls and seed testa [13]. In this study, expression results reflected rice GT8 genes with functional differentiation. Among them, three GT8 genes (*OsGATU7*, *OsGATL3*, and *OsGATL4*) had high expressions in anthers, implying that these three genes are involved in the anther development. *OsPGSIP-C1* and *OsGolS1* had high expression level in shoots and *OsGATL6* were highly expressed in seedling at the four-leaf stage, which suggests that *OsPGSIP-C1*, *OsGolS1*, and *OsGATL6* may be associated with shoots development and seedling growth. These findings were further supported by a recent experimental study that CRISPR-Cas9 knockout of *OsPGSIP-C1* led to dwarf plant height and spike shape changes of rice.

Earlier studies reported that GolS proteins play a key role in plant abiotic stress tolerances [12]. Cold stress and salt stress can lead to serious damage to crop growth and development, and can seriously affect the crop yield [36,38,51]. However, the potential roles of GT8 genes under abiotic stresses in Gramineae crops are poorly documented to date. Previously, orthologous groups were found to have similar function and gene structure, as well as conserved motifs [40,58]. We thus detected gene expression levels of *GT8* in response to salt and cold stresses and provided evidence that *OsGolS1* was significantly up-regulated under salt stress, implying *OsGolS1* plays an important role in salt stress tolerance. Additionally, *OsGAUT21*, *OsGATL2*, and *OsGATL5* were remarkably up-regulated under cold stress, which indicated that GT8 genes participate in rice cold stress tolerance. Taken together, these four genes and their orthologous in other tested Gramineae crops might be a potential for breeders to select candidate genes for mitigating plant stress.

## 5. Conclusions

In the present study, 269 GT8 genes were identified in Gramineae crop genomes, including 33, 37, 40, 41, 36, 37, and 45 GT8 genes in the genomes of *H. vulgare*, *B. distachyon*, *O. sativa* ssp. *japonica*, *O. rufipogon*, *S. italica*, *S. bicolor*, and *Z. mays*, respectively. Afterwards, we conducted comparative genomic and systematic analysis with respect to the phylogenetic relationship, chromosomal location, gene structure, protein motifs, promoter Cis-elements, orthogroups, duplication events, microsynteny relations, and expression patterns. Our results revealed that the expansion of the GT8 family might have occurred in multiple modes among these seven tested species. On the other hand, unequal losses of different orthogroups or subfamilies were found among them during the evolutionary process. Expression profiling and qRT-PCR results suggested that OsGT8 genes play an essential role in salt and cold stress. Furthermore, *OsGolS1*, *OsGAUT21*, *OsGATL2*, and *OsGATL5* may be excellent candidate genes for rice salt stress tolerance breeding.

## Supplementary Materials

The following are available online at https://www.mdpi.com/2218-273X/9/5/188/s1, Table S1: Primers of OsGT8 genes were used for qRT-PCR in this study. Table S2: The detailed information on GT8 genes in *H. vulgare*, *B. distachyon*, *O. sativa* ssp. *japonica*, *O. rufipogon*, *S. italica*, *S. bicolor*, and *Z. mays*. Table S3: Orthogroups among these seven species. Table S4: Number of orthogroups among these seven species. Table S5: Orthologues in *B. distachyon* with other six species. Table S6: Orthologues in *H. vulgare* with other six species. Table S7: Orthologues in *O. rufipogon* with other six species. Table S8: Orthologues in *O. sativa* ssp. *japonica* with other six species. Table S9: Orthologues in *S. bicolor* with other six species. Table S10: Orthologues in *S. italica* with other six species. Table S11: Orthologues in *Z. mays* with other six species. Table S12: Ka/Ks ratios and divergence times of duplicate gene pairs. Table S13: Transmembrane helical domains (TMHs) and subcellular localizations of GT8 proteins in *O. sativa* ssp. *japonica*. Table S14: statistical results of rice GT8 gene cis-element containing the information of Figure S4. Table S15: Pearson’s correlation coefficients of duplicate gene pairs. Figure S1: A high-resolution version of Figure 1. Figure S2: A high-resolution version of Figure 3. Figure S3: The phylogenetic tree (A), conserved motif compositions (B), and exon/intron structure (C) of the GT8 genes in these seven species. A. A UPGMA phylogeny tree of GT8 protein sequences from *H. vulgare*, *B. distachyon, O. sativa* ssp. *japonica*, *O. rufipogon*, *S. italica*, *S. bicolor*, and *Z. mays*. Different colors of branches represent different subfamilies. The different species are indicated by different shaped markers. B,C. The relative lengths of genes and proteins were showed by the widths of the grey bars. The exons and introns were displayed by yellow boxes and grey lines, respectively. Figure S4: Cis-acting elements in all GT8 genes of *Oryza sativa* ssp. *japonica*. A. The primary categories and secondary categories were shown by different bars and different characters. The different colors in histograms mean the number of different promoter elements in each secondary category. B. The different subfamilies of GT8 genes in the phylogenetic tree are shown by different colors and the differently colored boxes represent the different secondary categories. C. The ratios of primary/secondary categories are displayed by different sizes in pie charts. Figure S5: Co-expression values of all OsGT8 genes in all tissues (A), under cold stress (B), and under salt stress.

## References

[1] Lairson L.L., Henrissat B., Davies G.J., Withers S.G. (2008). Glycosyltransferases: Structures, functions, and mechanisms. Annu. Rev. Biochem..

[2] Sinnott M.L. (1990). Catalytic mechanisms of enzymatic glycosyl transfer. Chem. Rev..

[3] Coutinho P.M., Deleury EDavies G.J., Henrissat B. (2003). An evolving hierarchical family classification for glycosyltransferases. J. Mol. Biol..

[4] Vincent L., Hemalatha G.R., Elodie D., Coutinho P.M., Bernard H. (2014). The carbohydrate-active enzymes database (CAZy) in 2013. Nucleic Acids Res..

[5] Cosgrove D.J. (2005). Growth of the plant cell wall. Nat. Rev. Mol. Cell Biol..

[6] Yanbin Y., Huiling C., Hahn M.G., Debra M., Ying X. (2010). Evolution and function of the plant cell wall synthesis-related glycosyltransferase family 8. Plant Physiol..

[7] Kei-Ichiro I., Takako Y.M., Yuji H., Anderson M.E., Liping Y., Campbell K.P. (2012). Dystroglycan function requires xylosyl- and glucuronyltransferase activities of LARGE. Science.

[8] Sethi M.K., Buettner F.F.R., Angel A., Krylov V.B., Hideyuki T., Nifantiev N.E., Haltiwanger R.S., Rita G.S., Hans B. (2012). Molecular cloning of a xylosyltransferase that transfers the second xylose to O-glucosylated epidermal growth factor repeats of notch. J. Biol. Chem..

[9] Persson K., Ly H.D., Dieckelmann M., Wakarchuk W.W., Withers S.G., Strynadka N.C. (2001). Crystal structure of the retaining galactosyltransferase LgtC from *Neisseria meningitidis* in complex with donor and acceptor sugar analogs. Nat. Struct. Biol..

[10] Pitcher J., Smythe C., Cohen P. (2010). Glycogenin is the priming glucosyltransferase required for the initiation of glycogen biogenesis in rabbit skeletal muscle. Eur. J. Biochem..

[11] Atmodjo M.A., Yumiko S., Xiang Z., Burrell A.J., Mohanty S.S., Atwood J.A., Ron O., Scheller H.V., Debra M. (2011). Galacturonosyltransferase (GAUT)1 and GAUT7 are the core of a plant cell wall pectin biosynthetic homogalacturonan: Galacturonosyltransferase complex. Proc. Natl. Acad. Sci. USA.

[12] Taji T., Ohsumi C., Iuchi S., Seki M., Kasuga M., Kobayashi M., Yamaguchi-Shinozaki K., Shinozaki K. (2010). Important roles of drought- and cold-inducible genes for galactinol synthase in stress tolerance in *Arabidopsis thaliana*. Plant J..

[13] Caffall K.H., Pattathil S., Phillips S.E., Hahn M.G., Mohnen D (2009). *Arabidopsis thaliana* T-DNA mutants implicate GAUT genes in the biosynthesis of pectin and xylan in cell walls and seed testa. Mol. Plant.

[14] Chatterjee M., Berbezy P., Vyas D., Coates S., Barsby T. (2005). Reduced expression of a protein homologous to glycogenin leads to reduction of starch content in *Arabidopsis* leaves. Plant Sci..

[15] Huang J., Li J., Zhou J., Wang L., Yang S., Hurst L.D., Li W.-H., Tian D. (2018). Identifying a large number of high-yield genes in rice by pedigree analysis, whole-genome sequencing, and CRISPR-Cas9 gene knockout. Proc. Natl. Acad. Sci. USA.

[16] Jacquemin J., Ammiraju J.S., Haberer G., Billheimer D.D., Yu Y., Liu L.C., Rivera L.F., Mayer K., Chen M., Wing R.A. (2014). Fifteen million years of evolution in the Oryza genus shows extensive gene family expansion. Mol. Plant.

[17] Romani F., Reinheimer R., Florent S.N., Bowman J.L., Moreno J.E. (2018). Evolutionary history of HOMEODOMAIN LEUCINE ZIPPER transcription factors during plant transition to land. New Phytol..

[18] Haibao T., Bowers J.E., Xiyin W., Paterson A.H. (2010). Angiosperm genome comparisons reveal early polyploidy in the monocot lineage. Proc. Natl. Acad. Sci. USA.

[19] Vision T.J., Brown D.G., Tanksley S.D. (2000). The origins of genomic duplications in *Arabidopsis*. Science.

[20] Cedric S., Klaas V., Van Montagu M.C.E., Marc Z., Yves V.D.P. (2002). The hidden duplication past of *Arabidopsis thaliana*. Proc. Natl. Acad. Sci. USA.

[21] Bowers J.E., Chapman B.A., Junkang R., Paterson A.H. (2003). Unravelling angiosperm genome evolution by phylogenetic analysis of chromosomal duplication events. Nature.

[22] Olivier J., Jean-Marc A., Benjamin N., Alberto P., Christian C., Alberto C., Nathalie C., Sébastien A., Nicola V., Claire J. (2007). The grapevine genome sequence suggests ancestral hexaploidization in major angiosperm phyla. Nature.

[23] Yuannian J., Jingping L., Haibao T., Paterson A.H. (2014). Integrated syntenic and phylogenomic analyses reveal an ancient genome duplication in monocots. Plant Cell.

[24] Yan H.B., Pan X.X., Jiang H.W., Wu G.J. (2009). Comparison of the starch synthesis genes between maize and rice: Copies, chromosome location and expression divergence. Theor. Appl. Genet..

[25] Yu J., Wang J., Lin W., Li S.G., Li H., Zhou J., Ni P.X., Dong W., Hu S.N., Zeng C.Q. (2005). The Genomes of *Oryza sativa*: A history of duplications. PloS Biol..

[26] Zimmer E., Martin S., Beverley S., Kan Y., Wilson A.C. (1980). Rapid duplication and loss of genes coding for the alpha chains of hemoglobin. Proc. Natl. Acad. Sci. USA.

[27] Reed W.J., Hughes B.D. (2004). A model explaining the size distribution of gene and protein families. Math. Biosci..

[28] Hahn M.W., De B.T., Stajich J.E., Nguyen C., Cristianini N. (2005). Estimating the tempo and mode of gene family evolution from comparative genomic data. Genome Res..

[29] Conant G., Wolfe K. (2008). Turning a hobby into a job: How duplicated genes find new functions. Nat. Rev. Genet..

[30] Hideki I., Fyodor K. (2010). The evolution of gene duplications: Classifying and distinguishing between models. Nat. Rev. Genet..

[31] Xionglei H., Jianzhi Z. (2005). Rapid subfunctionalization accompanied by prolonged and substantial neofunctionalization in duplicate gene evolution. Genetics.

[32] Johnson D.A., Thomas M.A. (2007). The monosaccharide transporter gene family in *Arabidopsis* and rice: A history of duplications, adaptive evolution, and functional divergence. Mol. Biol. Evol..

[33] Force A., Lynch M., Pickett F.B., Amores A., Yan Y.L., Postlethwait J. (1999). Preservation of duplicate genes by complementary, degenerative mutations. Genetics.

[34] Lynch M., Conery J.S. (2000). The evolutionary fate and consequences of duplicate genes. Science.

[35] Lynch M., Force A. (2000). The probability of duplicate gene preservation by subfunctionalization. Genetics.

[36] Kong W., Zhong H., Gong Z., Fang X., Sun T., Deng X., Li Y. (2019). Meta-analysis of salt stress transcriptome responses in different rice genotypes at the seedling stage. Plants.

[37] Kong W., Zhong H., Deng X., Gautam M., Gong Z., Zhang Y., Zhao G., Liu C., Li Y. (2019). Evolutionary analysis of GH3 genes in six Oryza species/subspecies and their expression under salinity stress in *Oryza sativa* ssp. japonica. Plants.

[38] Cui S., Huang F., Wang J., Ma X., Cheng Y., Liu J. (2005). A proteomic analysis of cold stress responses in rice seedlings. Proteomics.

[39] Kong W., Yang S., Wang Y., Bendahmane M., Fu X. (2017). Genome-wide identification and characterization of aquaporin gene family in *Beta vulgaris*. PeerJ.

[40] Kong W., Bendahmane M., Fu X. (2018). Genome-wide identification and characterization of aquaporins and their role in the flower opening processes in carnation (*Dianthus caryophyllus*). Molecules.

[41] Shigehiro K., Zmasek C.M., Osamu N., Kazutaka K. (2013). aLeaves facilitates on-demand exploration of metazoan gene family trees on MAFFT sequence alignment server with enhanced interactivity. Nucleic Acids Res..

[42] Katoh K., Rozewicki J., Yamada K.D. (2017). MAFFT online service: Multiple sequence alignment, interactive sequence choice and visualization. Brief. Bioinform..

[43] Buchfink B., Xie C., Huson D.H. (2015). Fast and sensitive protein alignment using DIAMOND. Nat. Methods.

[44] Emms D.M., Kelly S. (2015). OrthoFinder: Solving fundamental biases in whole genome comparisons dramatically improves orthogroup inference accuracy. Genome Biol..

[45] Chen C., Xia R., Chen H., He Y. (2018). TBtools, a toolkit for biologists integrating various biological data handling tools with a user-friendly interface. BioRxiv.

[46] Wang Y.P., Tang H.B., DeBarry J.D., Tan X., Li J.P., Wang X.Y., Lee T.H., Jin H.Z., Marler B., Guo H. (2012). MCScanX: A toolkit for detection and evolutionary analysis of gene synteny and collinearity. Nucleic Acids Res..

[47] Krzywinski M., Schein J.I. (2009). Circos: An information aesthetic for comparative genomics. Genome Res..

[48] Librado P., Rozas J. (2009). DnaSP v5: A software for comprehensive analysis of DNA polymorphism data. Bioinformatics.

[49] Deng X., An B., Zhong H., Yang J., Kong W., Li Y. (2019). A novel insight into functional divergence of the MST gene family in rice based on comprehensive expression patterns. Genes.

[50] Magali L., Patrice D., Gert T., Kathleen M., Yves M., Yves V.D.P., Pierre R., Stephane R. (2002). PlantCARE, a database of plant cis-acting regulatory elements and a portal to tools for in silico analysis of promoter sequences. Nucleic Acids Res..

[51] Yun-Wei Y., Hung-Chi C., Wei-Fu J., Li-Yu L., Men-Chi C. (2015). Comparative transcriptome analysis of shoots and roots of TNG67 and TCN1 rice seedlings under cold stress and following subsequent recovery: Insights into metabolic pathways, phytohormones, and transcription factors. PLoS ONE.

[52] Vogel J.P., Garvin D.F., Mockler T.C., Schmutz J., Dan R., Bevan M.W., Barry K., Lucas S., Harmon-Smith M., Lail K. (2010). Genome sequencing and analysis of the model grass *Brachypodium distachyon*. Nature.

[53] Schulman A.H., Hastie A., Houben A., Chailyan A., Himmelbach A., Chapman B., Li C., Lin C., Colmsee C., Dockter C. (2017). A chromosome conformation capture ordered sequence of the barley genome. Nature.

[54] Bennetzen J.L., Schmutz J., Wang H., Percifield R., Hawkins J., Pontaroli A.C., Estep M., Feng L., Vaughn J.N., Grimwood J. (2012). Reference genome sequence of the model plant *Setaria*. Nat. Biotechnol..

[55] Paterson A.H., Bowers J.E., Rémy B., Inna D., Jane G., Heidrun G., Georg H., Uffe H., Therese M., Alexander P. (2009). The *Sorghum bicolor* genome and the diversification of grasses. Nature.

[56] Swigoňová Z., Lai J., Ma J., Ramakrishna W., Llaca V., Bennetzen J.L., Messing J. (2004). Close split of sorghum and maize genome progenitors. Genome Res..

[57] Coghlan A., Eichler E.E., Oliver S.G., Paterson A.H., Stein L. (2005). Chromosome evolution in eukaryotes: A multi-kingdom perspective. Trends Genet..

[58] Reuscher S., Akiyama M., Yasuda T., Makino H., Aoki K., Shibata D., Shiratake K. (2014). The sugar transporter inventory of tomato: Genome-wide identification and expression analysis. Plant Cell Physiol..

